# Finding the Balance to Quiet the Striving: The Difference Between Successful Aging and Wise Aging

**DOI:** 10.1093/geront/gnae126

**Published:** 2024-09-05

**Authors:** Judith Glück, Luisa Jäger, Irina Auer-Spath, Imke Alenka Harbig

**Affiliations:** Department of Psychology, University of Klagenfurt, Klagenfurt, Austria; Department of Psychology, University of Klagenfurt, Klagenfurt, Austria; Department of Psychology, University of Klagenfurt, Klagenfurt, Austria; Department of Psychology, University of Klagenfurt, Klagenfurt, Austria

**Keywords:** Aging, Self-transcendence, Successful aging, Wisdom, Wise aging

## Abstract

This paper draws on wisdom and lifespan development research to propose a conception of “wise aging,” which may become particularly relevant in very old age as people’s capacities for successful aging decline. We propose that 3 types of balance distinguish wise aging from successful aging. First, wisdom balances one’s own interest with the greater good, emphasizing self-transcendence and compassion. Second, wisdom balances control striving with acceptance of uncontrollability. Wise aging involves a realistic awareness of one’s decreasing levels of control and one’s interconnectedness to and dependence on other people. Third, wisdom acknowledges, regulates, and balances positive and negative affect. Wise aging involves the ability to appreciate and relish the joys of life, but also to accept and embrace more negative emotions and to support others going through different times.

The classical definition of successful aging includes three main components: “low probability of disease and disease-related disability, high cognitive and physical functional capacity, and active engagement with life” (Rowe & Kahn, 1997, p. 433). But even for people aging very successfully, aging always ends with death, often preceded by a period of decline in these three capacities. As people approach very old age, some shifts in focus may be useful for dealing with the challenges of the last life phase. This paper draws on wisdom research and lifespan psychology to propose three interrelated characteristics of wise aging. First, wise aging balances self-focused goals with goals aiming for the greater good. Second, wise aging balances control striving with acceptance of the limits of personal control. Third, wise aging embraces a broad spectrum of emotional experiences, balancing appreciation of joyful moments with acceptance of the darker sides of life. We first briefly review current psychological wisdom models and their relations to aging and then discuss the three components of wise aging in detail.

## What Wisdom Is and How It Is Related to Aging

The field of psychological wisdom research is growing fast and gaining public and academic interest. Until about 10 years ago, wisdom researchers tended to develop their own models and measures, which led to somewhat inconsistent evidence across different approaches. More recently, the state of research has been consolidating and some integrative models have been proposed. Table 1 gives an overview of the most influential cognitive-focused, personality-focused, and developmental conceptions of wisdom, the measures based on them, and their conceptual and empirical relations with aging (for detailed reviews, see e.g., Glück & Weststrate, 2022; Sternberg & Glück, 2022).

**Table 1.** Influential Models of Wisdom and Their Conceptual and Empirical Relationships With Aging

Wisdom model and measure(s)	Definition of wisdom + criteria/subcomponents	Conceptual relation with aging	Empirical relationships with aging
Cognitive-focused models			
Berlin Wisdom Model (Baltes & Smith, 1990; Baltes & Staudinger, 2000) Measure: Berlin Wisdom Paradigm (thinking-aloud interview about brief descriptions of difficult life problems; Staudinger et al., 1994)	Wisdom is expert knowledge about the “fundamental pragmatics of life,” that is, the important and difficult issues of human existence. Five criteria for wise responses: (a) factual knowledge and (b) procedural knowledge about life, meta-knowledge about (c) the uncertainty and unpredictability of life, (d) the differences between individuals in values and priorities, and (e) the influence of contexts on people’s thinking and behavior	Expert knowledge is obtained by people who engage in deliberate practice in the respective field. In these people, it increases or stays stable over the lifespan. Wisdom should grow or stay stable into old age in people who have wisdom-relevant personal traits, contexts, and experiences (Baltes & Smith, 1990)	Pasupathi et al. (2001) found a strong correlation between age and wisdom-related performance between ages 15 and 25 (Pasupathi et al., 2001); afterward, there were no age-related differences until about age 60 (Staudinger, 1999). Participants older than 60 tend to score lower than younger age groups (Ardelt, 2004; Staudinger, 1999), partly explained by declines in fluid intelligence (Glück & Scherpf, 2022).
Wise Reasoning Model (Grossmann et al., 2010; Oakes et al., 2019) Measures: (a) Written or spoken responses to vignettes about various types of interpersonal and social problems (e.g., Grossmann et al., 2010) (b) Situated Wise Reasoning Scale (SWIS): self-report scale about wise reasoning in past conflicts (Brienza et al., 2018)	Wise reasoning is a way of thinking about life problems that are characterized by (a) intellectual humility, (b) recognition of uncertainty and change, (c) consideration of others’ perspectives and broader context, and (d) searching for compromise and resolution	Components of wisdom that involve acknowledging different perspectives, searching for compromise, and recognizing the limits of knowledge should increase throughout the lifespan when assessed with context-rich, naturalistic materials (Grossmann et al., 2010).	Findings differ by measure: For responses to vignettes about intergroup and interpersonal conflicts, Grossmann et al. (2010) found increases in wise reasoning across three adult age groups. For self-reported wise reasoning in concrete situations, Brienza et al. (2018) found a U-shaped relationship with the lowest scores around age 45 (but few participants were older than 70).
Balance Theory of Wisdom (Sternberg, 1998, 2019) (no published measure)	Wisdom is practical intelligence applied to life problems with the aim of maximizing a common good. Wise solutions for complex life problems (a) balance the intrapersonal, interpersonal, and extrapersonal interests involved to find an ethical solution that maximizes a common good across all interests; (b) balance different ways to respond as required by the situation; and (c) balance consideration of short-term and long-term outcomes.	The idea of wisdom as practical intelligence implies that tacit knowledge plays a key role—knowledge that is acquired through experience or from role models and can be somewhat implicit. A willingness to think dialectically about problems can foster the development of wisdom, too (Sternberg, 2019).	(no empirical findings published)
Personality-focused models			
Three-Dimensional Wisdom Model (Ardelt, 2003; Ardelt et al., 2019) Measure: Three-Dimensional Wisdom Scale (3D-WS; Ardelt, 2003): 39-item self-report scale measuring the reflective, cognitive, and compassionate personality dimension.	Wisdom is a personality quality that combines (a) a reflective dimension (habitually taking different perspectives on life problems); (b) a cognitive dimension (a desire to understand phenomena and events, especially intrapersonal and interpersonal matters); and (c) a compassionate dimension (positive emotions and caring concern toward others).	No specific predictions concerning aging. Generally, Ardelt considers the reflective dimension as a predecessor of development in the other dimensions (Ardelt, 2003; Ardelt et al., 2019)	Scores in the 3D-WS are typically negatively correlated with age (Ardelt, 2003; Glück, 2019; Glück et al., 2013). In a large lifespan sample, scores on the cognitive dimension were highest between 40 and 60 and declined markedly from age 60 on, whereas the reflective and affective dimensions had a U-shaped relationship with age, with the highest scores in the oldest age groups (Ardelt et al., 2018).
Self-Transcendence Model of Wisdom (Aldwin et al., 2019; Levenson et al., 2005) Measure: Adult Self-Transcendence Scale (ASTI; Koller et al., 2017; Levenson et al., 2005): 34-item self-report scale measuring self-transcendence. Koller et al. (2017) identified five subdimensions.	Wisdom is self-transcendence: feeling connected to other people, past and future generations, and nature; having a sense of meaning in life and often experiencing positive emotions such as joy, inner peace, and awe. Self-transcendence “is the antithesis of the narcissism and materialistic strivings which are so often at the heart of psychological distress” (Aldwin et al., 2019, p. 137).	Levenson et al. (2005) conceptualized four components of self-transcendence as loosely developmentally ordered: self-knowledge, detachment, integration, and self-transcendence (pp. 128–129).	In Koller et al.’s (2017) study, participants older than 31 (but mostly younger than 60) scored higher than younger participants in nonattachment and self-transcendence; there were no age differences in the other three subscales (self-knowledge and integration; peace of mind; and presence in the here-and-now and growth).
Developmental models			
HERO(E) Model of Wisdom (Webster, 2003, 2007) Measure: Self-Assessed Wisdom Scale (SAWS; Webster, 2007): 40-item self-report scale measuring the five subdimensions.	Wisdom is the ability to apply insights gained from life experiences to facilitate the optimal development of oneself and others. It involves (a) critical life experience, (b) openness, (c) reminiscence and reflectiveness, (d) emotional regulation, and (e) humor.	Based on models of fluid and crystallized intelligence, Webster et al. (2014) argued that wisdom should first increase as life experiences are accumulated, peak in midlife, and then start declining as limitations in cognitive, physical, and social resources increase.	Webster et al. (2014) found an inverse U-shaped relationship between wisdom and age in a lifespan sample, with scores in most of the five components declining after age 60.
MORE Life Experience Model (Glück & Bluck, 2013; Glück et al., 2019) Measure: MORE Life Experience Interview (Glück et al., 2019): interview about autobiographical life challenges.	Life challenges are catalysts for the development of wisdom. Five psychological resources predict whether people gain wisdom from life challenges: (a) managing uncertainty and uncontrollability; (b) openness to new perspectives and tolerance of differences; (c) reflectivity; (d) emotional sensitivity and (e) emotion regulation (with respect to self and others).	The MORE resources are assumed to predict what life challenges people encounter, how they deal with them, and what insights they gain as they reflect on challenges.	No findings published on age differences yet.

As Table 1 shows, cognitive-focused models define wisdom as a way of thinking about life problems that is acquired through experience and reflection. Wisdom-related thinking is characterized by both extraordinary knowledge and extraordinary awareness of the limitations of that knowledge, the uncertainty and uncontrollability of life, and the multiperspectivity of difficult life problems (Baltes & Staudinger, 2000; Oakes et al., 2019; Sternberg, 2019). Personality-focused models complement cognitive models by focusing on personality characteristics such as a willingness to take different perspectives, compassionate concern for others, self-transcendence, and inner peace (Aldwin, 2019; Ardelt, 2003). Developmental models focus on how wisdom develops. They assume that life challenges are necessary for the development of wisdom, but characteristics such as openness, reflectivity, or emotion regulation influence whether people actually gain wisdom from challenges (Glück et al., 2019; Webster, 2007).

Recently, wisdom research has entered a new era of integration, as several integrative conceptions have been proposed that combine elements from different models. These conceptions are summarized in Table 2. In sum, wisdom is a complex and multifaceted construct, and different models have focused on different components. Recent integrative accounts demonstrate, however, that the different models are compatible rather than contradictory (Glück & Weststrate, 2022).

**Table 2.** Integrative Models of Wisdom

Authors and model	Approach and components
Jeste & Lee (2019)	Identified cIdentified components of wisdom models that are consistent with neurobiological, developmental, and evolutionary perspectives: Wisdom is “a complex human trait with several specific components: social decision making, emotional regulation, prosocial behaviors such as empathy and compassion, self-reflection, acceptance of uncertainty, decisiveness, and spirituality”
Karami et al. (2020): Polyhedron model	Reviewed wisdom conceptions from the fields of psychology, management, and education and identified three cognitive components (knowledge management; sound judgment and decision-making; and intelligence and creative thinking) and three noncognitive components (self-regulation; openness and tolerance; and altruism and moral maturity).
Grossmann et al. (2020): Common-denominator model	Conducted an expert survey to identify elements of wisdom on which the experts agreed. The resulting common-denominator model includes two broad components: perspectival meta-cognition (balancing different viewpoints, epistemic humility, context adaptability, and multiple perspectives) and moral aspirations.
Glück & Weststrate (2022): “elephant model” of wise behavior in difficult life situations.	Three broad noncognitive components (an exploratory orientation, concern for others, and emotion regulation) predict whether people are able to maintain a “wise” state of mind even in challenging situations, which enables them to fully access their cognitive wisdom capacities (knowledge about life and oneself, metacognitive capacities, and self-reflection).

### Wisdom and Aging

Different components of wisdom show different developmental trajectories over the lifespan. Sternberg (2005) reviewed different views of how wisdom might be related to age but then argued that “individual differences in the development of wisdom are so large that averages probably tell us little about how wisdom develops. It is not any kind of experience (i.e., crystallized intelligence) in itself that leads to wisdom, but rather the decision to use that experience in a reflective, action-oriented way that leads to a common good” (Sternberg, 2005, p. 13). This view is consistent with developmental conceptions of wisdom, which argue that how people reflect on experiences is more important than the nature of the experiences in itself.

In sum, different people show different developmental patterns with respect to wisdom, and cross-sectional relationships with age do not tell us much of the story of how wisdom develops. At the same time, there is some convergence across measures of wisdom. An inverse U-shaped relationship with aging, with the highest scores between about age 40 and 60, has been found for three different wisdom measures (the Three-Dimensional Wisdom Scale, Ardelt et al., 2018; the Berlin Wisdom Paradigm, Staudinger, 1999; and the Self-Assessed Wisdom Scale, Webster et al., 2014), although other measures show different patterns, such as a linear increase with age for the Wise Reasoning Paradigm (Grossmann et al., 2010) or a U-shaped relationship for the Situated Wise Reasoning Scale (Brienza et al., 2017). Glück (2019) argued that the different patterns can be explained by the components of wisdom that the measures emphasize. An aspect of wisdom that would seem particularly likely to decline with aging, due to declines in fluid intelligence, is multi-perspectival reasoning about complex theoretical life problems. This may explain why both performance in the Berlin Wisdom Paradigm and the cognitive dimension of the Three-Dimensional Wisdom Scale (i.e., self-reported willingness to engage in complex thinking) are negatively related to age in older adults (e.g., Ardelt, 2003; Kunzmann, 2018; Staudinger, 1999). Other components of wisdom, however, have been found to increase well into advanced old age. These components include considering others’ perspectives and searching for compromise (Grossmann et al., 2010), compassionate love for others (Ardelt et al., 2018), and self-transcendence (Glück, 2019; Koller et al., 2017). These changes are at the heart of the model of wisdom as self-transcendence proposed by Aldwin et al. (2019), Igarashi et al. (2018), and Levenson et al. (2005). Self-transcendent individuals feel connected to other people, past and future generations, and nature. They have a sense of meaning in their life and often experience positive emotions such as joy, inner peace, and awe. The self-transcendence model builds on earlier research on gerotranscendence (Tornstam, 1994) showing that in advanced old age, many people experience an increased connectedness to past and future generations and humanity in general, decreased fear of death, and reduced self-centeredness and dependence on external sources of self-esteem. Aldwin et al. (2019) reviewed research showing positive relationships between transcendence and well-being and mental health in very old people and people with serious health conditions. Self-transcendence has been found to increase across old age. In a meta-analysis of wisdom measures, Dong et al. (2023) found a significant relationship between self-transcendence, as measured by the Adult Self-Transcendence Inventory, and age (*r* = 0.096, *p* = .003).

To summarize, high levels of wisdom are, unfortunately, rare at any age. Most people gain some insights about life over time, but few people seem to gain insights that make them wiser. At the same time, some components of wisdom seem to be quite prevalent in advanced old age. These aspects may become more important as sources of well-being when other sources, such as control and success, become less available.

## Conceptualizing Wise Aging

Based on theoretical considerations and empirical findings from wisdom research, we propose three components of wise aging that may be conducive to well-being in very old age. Although many people may age successfully in the classical sense for a long time, at some point losses in the domains of health, cognition, active engagement, and social contacts begin to accumulate. Late-life decline is different from difficult phases earlier in life in that the future is likely to bring even more decline and it always ends with death. Clearly, growing old is indeed “not for sissies” and some resources that support successful aging in earlier phases may be less adaptive in very old age.

We propose that wise aging involves three interrelated aspects. First, a shift toward more self-transcendent goals creates new sources of well-being beyond personal goal attainment. Second, and relatedly, awareness and acceptance of one’s own decreasing control and capacities can foster experiences of interconnectedness, gratitude, and relishing. Third, embracing a broader spectrum of emotional experiences may be more adequate for dealing with advanced old age than a focus on positive affect achieved through suppression and distraction. In the following, we discuss these three components in detail. Each component will be illustrated by quotes from our first study of wisdom development, where 94 participants, 50% of them wisdom nominees, were interviewed about difficult events from their lives (see e.g., Glück et al., 2013; König & Glück, 2014; Weststrate & Glück, 2017a). The quotes are translated from Glück (2016); some were also cited in Glück (2022). As the sample of that study covered a broad age range, most quotes come from participants younger than very old age, but we believe they still illustrate the relevant facets of wise aging well.

### Shifting Toward Self-Transcendence: Balancing Self-Focused Goals With a Greater Good

*I have developed a feeling of compassion for this whole complex of judgment and condemnation that’s going on between people […]. I’ve come to see the pain in judging and in being judged very clearly.* (woman, 47 years)

*I will never demand anything from my children. […] My children know that as long as I can live and think on my own, I’ll be living alone. If that’s not possible anymore, they can place me in a nursing home, even if I perhaps refuse out of confusion. The neighbors know that too, so that no one can say, now they’re putting their poor old mama in a home. Because I think the children have to think forward, to care about their own families, just as I cared about my families, not about the old. The old get buried. And children don’t need to be grateful. What should they be grateful for? That they were born? They didn’t ask to be born. We wanted them. We enjoyed them. They didn’t always enjoy us.* (woman, 92 years)

The classical definition of successful aging does not imply particular goals, except for an implicit focus on maintaining one’s own health and cognitive and physical capacities. However, there is a strong body of research showing that pursuing altruistic goals, such as volunteering, is conducive to well-being in old age (e.g., Kahana et al., 2013; Sin et al., 2021), and several authors have suggested to include generativity or prosociality as characteristics of successful aging (e.g., Becchetti & Bellucci, 2021; Hämäläinen et al., In press; McCarthy et al., 2013). Broader conceptions of transcendence as a characteristic of late life typically include components of greater-good engagement such as generativity and altruism (e.g., McCarthy & Bockweg, 2013). Thus, although the term “successful aging” does not necessarily imply any type of “success” beyond one’s own personal goals, it has often been demonstrated that engaging oneself for a greater good is conducive to well-being in any life phase and perhaps particularly so in old age.

Wisdom is broadly associated with non-egoistic, other-oriented goals. Engaging oneself for others is a typical characteristic in folk conceptions of wisdom (see e.g., typical wisdom exemplars such as Mohandas Gandhi, Mother Teresa, or Jesus Christ; Paulhus et al., 2002; Weststrate et al., 2016). A greater-good orientation is also, explicitly or implicitly, part of most psychological wisdom models. In Sternberg’s balance theory, making ethical decisions to maximize a common good is key to wisdom (Sternberg, 2019). The common-factor model of wisdom (Grossmann et al., 2020) also includes an ethical orientation; the Three-Dimensional Wisdom Model (Ardelt, 2003) and the MORE Life Experience Model (Glück et al., 2019) include components of compassion and concern for others. The self-transcendence model explicitly builds on research suggesting that overcoming ego-based strivings, feeling deeply connected to others, and experiencing oneself as part of something greater can help individuals cope with illness, loss, and the impending end of life (e.g., Bauer & Wayment, 2008; Tornstam, 1994).

Wise aging does not mean completely giving up self-related goals, but one’s personal benefit becomes less important in relation to the greater good. Wise individuals might, for example, experience joy at the successes of others without wanting credit for their own contribution. Wise aging also balances short-term and long-term goals (Sternberg, 1998, 2019). Socioemotional selectivity theory has demonstrated that decreasing perceived lifetime increases a focus on close relationships and positive affect (Carstensen, 2021); self-transcendence may add larger goals beyond the end of one’s own life (Aldwin et al., 2019). According to Erikson’s theory of identity development, a growing focus on contributing to a greater good beyond one’s personal life is characteristic of middle age (Erikson, 1980). In sum, goals that transcend personal achievements are less vulnerable to losses in personal resources and capacities. In fact, the positive effects of a shift from self-interest toward broader interests may not be limited to advanced old age (Wayment & Bauer, 2008; see also Bauer & Weatherbie, 2023; Wayment & Bauer, 2018) and such shifts on a global scale may also be crucial to the survival of our species.

### Balancing Control Striving With Awareness of Uncontrollability

*Of course, there’s always a bit of sadness involved. But I’m feeling better now, and that’s because I’m taking the pressure off myself, the pressure of wanting something. Because I won’t get it. Because my mother cannot give it to me. And so I’ve come to accept this and say, I’m happy about what I do get, and I accept it gratefully, and more is not going to happen. […] I think it’s important to forgive. To somehow come to terms with the situation, to try to understand the other person, or aspects of the other person and her life, and then you can say: Yes. I don’t like the way it is, but I accept it.* (woman, 40 years)

*Suddenly something happens that no one foresaw. And there’s nothing you can do about it. It’s just there, all of a sudden. And for me, the point is that I have come to relish every day; I’m seventy now and more or less healthy and all, but I know, of course, that it’s not going to stay that way. This awareness that things are not going to exist anymore, that may have been the deepest cut in my living, my thinking, my everyday life.* (man, 71 years)

Psychological conceptions of successful aging tend to emphasize the maintenance of subjective control as an important source of well-being, even in the face of losses in objective control (e.g., Baltes & Baltes, 1990; Heckhausen et al., 2019). Research has shown that perceived control is positively related to well-being and suggested that high levels of perceived control should be maintained as long as possible (e.g., Robinson & Lachman, 2017). For example, increasing clients’ control in caregiving settings has very important positive effects (Mallers et al., 2014). At the same time, both objective and perceived control decline with age, especially as people approach the end of their lives (e.g., Antonides & van Wijngaarden, in press; Drewelies et al., 2017; Pickard, In press). According to lifespan theories of control (e.g., Heckhausen et al., 2019), when goals become objectively unattainable, strategies such as compensatory secondary control (giving up goals) are considered adaptive. High perceived control is less adaptive with respect to goals that are, in fact, uncontrollable. Perceived control over uncontrollable future stressors is actually negatively, not positively, related to well-being (Frazier et al., 2011), and acceptance of uncontrollability would seem more adaptive (e.g., Broadbent et al., 2014). Such stressors, such as unpredictable changes in one’s health or loss of close others, would seem quite typical for very late life. In our view, acceptance of uncontrollability is more than just giving up striving for a goal. It may be part of a broader picture of reduced striving for self-focused goals and more relishing of positive present moments. Wisdom entails an awareness and acceptance of what can and cannot be controlled (as in the classical “serenity prayer”) at any age. In fact, experiences of uncontrollability are considered to be important possible catalysts for growth in wisdom (e.g., Glück et al., 2019). The Berlin Wisdom Model and the Wise Reasoning Model include awareness of uncertainty and epistemic humility as components, and the MORE Life Experience Model views the management of uncertainty and uncontrollability as a developmental wisdom resource. The self-transcendence model draws on Buddhism in proposing nonattachment (to external sources of self-esteem such as power and achievement) as a core component of wisdom (Aldwin et al., 2019). Wise individuals know what they can and what they cannot control; and while they certainly will engage themselves for reachable goals (e.g., through health behaviors), they also know that engagement does not guarantee success.

Awareness of limited control implies awareness of one’s dependence on others. The self-transcendence model in particular proposes that being aware of the interconnectedness and interdependence among human beings is an important part of wisdom. Wisdom is positively related to gratitude (König & Glück, 2014), and requesting and/or accepting emotional support is an important resource of growth in wisdom from negative experiences (Igarashi et al., 2018).

In sum, consistent with the reduction in ego-focused goals, wise aging also entails a reduction in perceived control. Individuals aging wisely are aware of the decreases in their cognitive and physical capacities and the decreases in control that they bring with them, but they are able to accept and embrace these losses. Their sense of self is not dependent on a sense of control, and they feel close enough to other people to trust that they will not be alone, whatever happens. For people who tend to draw their self-esteem from achievement, this may mean a rather fundamental shift from feeling respected to feeling loved.

### Balancing Joy and Happiness With Awareness of the Dark Sides of Life

*The most important thing I’ve learned is to accept myself. Because I had weaknesses on so many levels that I was first ignoring and then hiding, from others and from myself, I got into an enormous self-betrayal, a self-lie. I remember I was told that someone was envious. Ha, envy? I don’t know that feeling! […] But over time I learned to follow my feelings, to get to the bottom of them. I recently met a lady who said her husband had died half a year ago, so she had just spent six weeks in the U.S. to distract herself. That made me think if my husband dies, the man I’ve loved, the man I was so attached to, then I won’t go distracting myself. I’ll enter into this process of grief, and I’ll bear it with dignity.* (woman, 65 years)

*Two days ago, the view from this window was so beautiful –the sun, the snow, the mountains, it’s just such a joy to see. It gives you such deep gratitude.* (woman, 92 years)

The use of well-being as the main indicator of successful aging has long been criticized (see e.g., Freund & Riediger, 2003), but researchers still tend to emphasize high well-being and positive affect as important outcomes of successful aging. Research based on Socioemotional Selectivity Theory (e.g., Carstensen, 2021) has broadly demonstrated that older adults aim to maximize positive affect and minimize negative affect, focusing on positive experiences as they perceive their remaining lifetime as limited. According to the Strength and Vulnerability Integration model of changes in emotion regulation across adulthood, a stronger physical reaction to negative emotions, if they do occur, is another reason why older people tend to avoid negative experiences (Charles, 2010). A more recent focus in emotion research suggests, however, that at least in some cases acceptance, rather than avoidance or reappraisal, of negative emotions may be beneficial to well-being and tends to increase with age (Mahlo & Windsor, 2021; Shallcross et al., 2013; Wolfe & Isaacowitz, 2021). For example, Shallcross et al. (2013) found that older age was associated with higher acceptance and lower anger and anxiety (but not sadness) across different measures, and acceptance statistically mediated the relationship between age and both anger and anxiety. Research on mindfulness has also found that acceptance of negative feelings as they are can reduce their effects on general well-being (Mahlo & Windsor, 2021). Generally, although in “Western” cultures, negative emotions are typically perceived as undesirable, “Eastern” cultures tend to have a more holistic understanding of affect, with a view of positive and negative emotions as covarying and often mixed, which has been linked to a lower prevalence of affective disorders (De Vaus et al., 2017).

Wisdom tends to manifest itself most clearly in emotionally challenging situations (Glück et al., 2005; Glück & Weststrate, 2022), and several researchers consider the ability to deal with negative emotions as central to wisdom. Webster’s HERO(E) Model of wisdom includes both emotion regulation, the ability to understand and regulate one’s own and other’s emotions, and humor, the ability to cope with challenges and support others through being aware of the ironies of life and being able to chuckle at one’s own failures (Webster, 2003, 2007). The MORE Life Experience model includes components of emotional sensitivity and emotion regulation (Glück et al., 2019). Thus, wisdom helps people deal with the negative emotions that challenging situations bring about. Because of this focus on difficult situations, there has been a debate among wisdom researchers about whether wiser individuals are happier or perhaps even less happy than other people. Some authors, most prominently Monika Ardelt, have argued that an ideally wise person has developed a “more profound sense of well-being through the development of equanimity, gratitude, and acceptance” (Ardelt, 2019, p. 602). Other authors, however, believe that wisdom should not be positively related to well-being because it entails an emotional confrontation with the dark and negative aspects of human existence (e.g., Staudinger & Glück, 2011). According to current emotion psychology, negative emotions have adaptive functions and contain important information (Kunzmann & Glück, 2019). For example, Kunzmann & Wrosch (2024) argued that anger and sadness are differentially adaptive in different life phases. Anger is highly adaptive for young adults, motivating them to take control and overcome obstacles. Sadness, on the other hand, is more adaptive in advanced old age when people need to grieve and accept irredeemable losses.

We propose that wise individuals at any age indeed experience high levels of well-being (Glück & Scherpf, 2022), but wise aging also entails experiencing and acknowledging negative emotions as wise individuals are aware of the full spectrum of human experience. They are willing to support others going through negative experiences and to reflect on the meaning of their own sadness, grief, or anger (Labouvie-Vief, 2003). Grossmann et al. (2019) demonstrated that wise reasoning is related to emodiversity—recognizing and balancing a range of emotions—rather than to downregulating emotions in general.

Weststrate and Glück (2017b) gave three reasons why wise individuals are able to maintain high levels of well-being even though they embrace the darker sides of life. First, wise people are experts at dealing with difficult situations, having the experience, emotion regulation capacities, and coping skills necessary for navigating their own and other people’s challenges. Second, wise people appreciate and relish the positive sides of life (Beaumont, 2011; König & Glück, 2014). Third, wise people know what helps them to maintain their emotional balance (such as close relationships, contact with nature, or art and literature), and they make sure to have access to those resources (Weststrate & Glück, 2017b). In sum, although direct evidence is limited, we propose that wise individuals, at any age, embrace the full spectrum of human emotions because they can, due to their abundant resources for emotion regulation and well-being. This ability makes them excellent supporters for others who are suffering. They do not suppress negative emotions but acknowledge them, as they consider them as important. They do not, however, exacerbate or dwell on them in ways that are harmful to themselves or others.

## Discussion and Outlook

The goal of this paper was to propose a conception of wise aging that is not incompatible with conceptions of successful aging but may be more suitable for navigating the challenges and demands of very old age, when successful aging may no longer be possible. From a medical and public health perspective, extending the period of successful aging, where people have sufficient cognitive and physical capacities for independent living and life engagement, is a highly valuable goal. Still, even highly successful aging inevitably ends with death, and death tends to be preceded by an extended period of not-so-successful aging. Our review of the literature suggested that some components of wisdom may be particularly conducive to navigating this late phase of life well: a balance between self-focused goals and greater-good goals, a balance between striving for control and accepting uncontrollability, and a balance between relishing positive times and accepting and embracing painful and sad times.

The components of wise aging that we have identified draw most closely on the conception of gerotranscendence (Tornstam, 1994; overview in Aldwin et al., 2019). As personal resources decline, a quieter ego (Wayment & Bauer, 2008) can help people focus on priorities beyond their personal situation. Self-transcendent emotions such as gratitude and awe involve an awareness of the relative unimportance and tininess of oneself in the greater scheme of things, which can come as a relief when one’s powers are declining. Conceptions of aging as a continuous striving for the maintenance of performance levels may be more reflective of young to middle-aged researchers’ ideas of aging. Even purposeful striving for wisdom may no longer be a priority in very old age. As Erik Erikson put it when he took a late-life perspective on his own work, “The demand to develop Integrity and Wisdom in old age seems to be somewhat unfair, especially when made by middle-aged theorists - as, indeed, we then were” (Erikson, 1984, p. 160).

It is an interesting question how people arrive at the three types of balance that characterize wise aging. Generally, developmental models of wisdom propose that people who are open to personal growth, interested in the difficult questions of life, and highly self-reflective are more likely to grow wiser in the course of their lives by gaining insights about the importance of a greater good, the limitations of personal control, and the full spectrum of emotional experience (Glück & Bluck, 2013; Glück et al., 2019; Webster, 2003, 2007). The ideas laid out in this paper suggest, however, that the experiences of very old age may foster these components of wisdom even in people who have not been on the “wisdom track” for most of their lives. Experiencing losses in health and personal control and awareness of one’s proximity to death may lead to an increased awareness of the importance of connectedness to others, the uncontrollability of much of our existence, and the complexity of the emotions associated with looking back, but also the importance of relishing the joys of life (Erikson, 1984; Kinnier et al., 2001).

It is important to note that a person’s opportunities for wise aging (as well as for successful aging) may be strongly dependent on social and socioeconomic resources. For example, someone spending their last years of life in an understaffed and underfunded nursing home with little social support or even contact will not be in a position to gain wisdom from experiencing connectedness to others; the limitations of personal control and the presence of negative feelings such as sadness and loneliness may be painfully obvious to them but unlikely to lead to a stance of acceptance and peace of mind. It seems important to consider what conditions are necessary to enable people to age wisely. A caring, respectful, and validating social environment would seem highly important for helping people navigate the transitions of late life.

In addition to contributing to personal well-being in advanced old age, a greater focus on the three components of wise aging in *all* life phases might have benefits for the future of humanity. Conceptions of good aging reflect, to some extent, societal goals and expectations. Conceptions of “successful” aging were conceived during a time of (at least in the global North) peace and security, economic growth and increasing prosperity, and a positive outlook on the future. In turn, psychological conceptions of successful aging focused on the maintenance of subjective control, achievement of personal goals, and well-being for as long as possible. The 21st century has brought along major changes. The growing threat of climate change and its consequences in combination with global and national inequality, polarization, and radicalization fired up by the algorithms and feedback systems of social media, and the collective experience of a global pandemic have created a world of uncertainty and uncontrollability. In addition, as Bauer and Wayment (2008) have argued, our modern world tends to reinforce excessive self-interest. The growing rate of mental illness in many countries may be partly a cause and partly a consequence of the dire outlook on our future. If we could foster even a small shift of people’s priorities and orientations in the direction of the components of wise aging proposed in this paper, we might have a better chance of survival.

## Data Availability

This manuscript does not report any data; therefore, the pre-registration and data availability requirements are not applicable.
